# Ranolazine in Chronic Stable Angina: A Systematic Review and Meta-Analysis

**DOI:** 10.7759/cureus.115878

**Published:** 2026-09-07

**Authors:** Peter Collins, Waleed Alhabeeb, Imad Alhaddad, Abdulhalim J Kinsara, Khalid Al Faraidy, Mohammed Balghith, Hany Ragy, Wafa Fehri, Iliyasse Asfalou, Fakhr Al Ayoubi, Hazem Amer, Duraid Al-Rammahy, Nancy Batah

**Affiliations:** 1 Clinical Cardiology Department, National Heart and Lung Institute, Imperial College London, London, GBR; 2 Cardiac Sciences Department, King Saud University, Riyadh, SAU; 3 Cardiovascular Department, Jordan Hospital, Amman, JOR; 4 Cardiology Department, King Saud Bin Abdulaziz University for Health Sciences College of Medicine, Ministry of National Guard Health Affairs, King Abdullah International Medical Research Center, Jeddah, SAU; 5 Interventional Cardiology Department, King Fahad Military Medical Complex, Dhahran, SAU; 6 Interventional Cardiology Department, King Abdulaziz Medical City, Riyadh, SAU; 7 Cardiology Department, National Heart Institute, Cairo, EGY; 8 Cardiology Department, Military University Hospital, Tunis, TUN; 9 Cardiology Department, Mohammed V Military Hospital, Mohammed V University, Rabat, MAR; 10 Cardiac Sciences Department, King Fahad Cardiac Center, Riyadh, SAU; 11 Otorhinolaryngology Department, Mansoura University, Mansoura, EGY; 12 Medical and Scientific Affairs Department, Menarini Middle East and Africa FZ LLC, Dubai, ARE

**Keywords:** angina, cardiovascular disease, chronic stable angina, myocardial infarction, ranolazine

## Abstract

Chronic stable angina (CSA) significantly impairs functional capacity and quality of life (QoL), with many patients remaining symptomatic despite standard treatment. Ranolazine, a late sodium current inhibitor, is utilized as an adjunctive antianginal therapy. This systematic review and meta-analysis evaluates its efficacy and safety in adult patients with CSA. We searched major databases through April 2025 for studies comparing ranolazine to placebo or standard therapy. Efficacy outcomes included angina burden, exercise parameters, QoL, and major adverse cardiovascular events (MACE), including myocardial infarction (MI) and revascularization. Safety was assessed via adverse event incidence. Data were synthesized using random-effects meta-analysis. Sixty-nine studies were included. Ranolazine significantly improved objective exercise measures, increasing exercise duration (mean difference {MD}, 33.1 seconds; 95% confidence interval {CI}, 27.3-38.9), time to angina onset (MD, 42.8 seconds; 95% CI, 34.2-51.5), and time to 1-mm ST depression (MD, 43.2 seconds; 95% CI, 33.8-52.6). Ranolazine was associated with a reduced risk of MI (risk ratio {RR}, 0.91; 95% CI, 0.84-0.98), coronary artery bypass grafting (CABG; RR, 0.55; 95% CI, 0.35-0.86), and any revascularization such as percutaneous coronary intervention (PCI) or CABG (RR, 0.77; 95% CI, 0.64-0.91). Adverse events were more frequent in the ranolazine group (RR, 1.45; 95% CI, 1.27-1.66) but were predominantly non-serious. Ranolazine provides robust symptomatic and functional benefits in patients with CSA. It is associated with modest reductions in MI and revascularization procedures, such as PCI or CABG. The data support ranolazine as an effective therapy for patients with CSA.

## Introduction and background

Chronic stable angina (CSA) remains a significant global health concern and is one of the most common clinical manifestations of coronary artery disease (CAD) [1]. It is characterized by episodes of chest discomfort or pain triggered by emotional stress or physical exertion, typically relieved by rest or the administration of short-acting nitrates. Although not immediately life-threatening, CSA imposes a substantial burden on patients’ daily functioning, quality of life (QoL), and psychological well-being [1,2]. The effective management of angina is crucial for reducing symptom burden, improving functional capacity, and potentially preventing the progression of ischemic heart disease.

Ranolazine, a novel antianginal agent, acts by selectively inhibiting the late sodium current in myocardial cells, thereby reducing intracellular calcium overload and improving myocardial relaxation and perfusion [3]. Unlike traditional antianginal drugs, ranolazine does not significantly affect heart rate or blood pressure, making it a potentially attractive option, particularly for patients who are intolerant to or inadequately controlled by conventional therapies [4]. Over the past two decades, several studies (including trials and observational studies) have evaluated the efficacy and safety of ranolazine in CSA, using a range of clinical and patient-reported outcomes such as exercise tolerance, the frequency of angina episodes, nitrate use, Seattle Angina Questionnaire (SAQ) scores, and major adverse cardiovascular events (MACE). Nevertheless, variability persists in the reported effectiveness of ranolazine, with differences in study designs, follow-up durations, population characteristics, and outcome measures. While several studies suggest that ranolazine offers meaningful benefits in improving angina symptoms and functional parameters, others report modest or inconclusive results. Additionally, some studies report outcomes narratively or present data in formats that preclude quantitative synthesis, highlighting the need for a comprehensive assessment [5].

Therefore, we conducted this systematic review and meta-analysis to evaluate the safety and efficacy of ranolazine in adult patients with CSA. By integrating evidence from randomized trials, non-randomized studies, and real-world data, we provide the most comprehensive assessment to date of its impact on clinical and patient-centered outcomes, aiming to clarify its role in the management of CSA and to inform clinicians, researchers, and policymakers in evidence-based decision-making.

## Review

Methods

We adhered to the PRISMA 2020 reporting standards in conducting and presenting this systematic review and meta-analysis [6]. The protocol was prospectively registered with the International Prospective Register of Systematic Reviews (PROSPERO) (CRD420251051059).

Eligibility Criteria

We included original studies involving adult patients (≥18 years) with CSA that quantitatively evaluated the use of ranolazine compared to standard therapy, placebo, or other antianginal agents. Eligible studies assessed outcomes that captured reductions in symptom burden (e.g., SAQ scores and the use of short-acting nitrates), improvements in functional capacity (e.g., exercise duration and time to angina), and the prevention of ischemic heart disease progression (e.g., the incidence of MACE, myocardial infarction {MI}, percutaneous coronary intervention {PCI}, and coronary artery bypass grafting {CABG}). Safety outcomes of ranolazine included the incidence of adverse events. We included randomized controlled trials (RCTs), non-randomized interventional studies, and observational studies (cohort or case-control designs). Studies without extractable quantitative data (e.g., lacking measures of effect, standard deviations, or sample sizes) were included in the narrative synthesis only, provided they reported relevant outcomes. We excluded studies involving pediatric populations, animal or in vitro studies, reviews, case reports, editorials, and conference abstracts lacking full data.

Search Strategy

A systematic search of Medical Literature Analysis and Retrieval System Online (MEDLINE) (via PubMed), Excerpta Medica database (EMBASE), Web of Science, and the Cochrane Central Register of Controlled Trials (CENTRAL) was conducted from inception to April 2025. Additional sources included ClinicalTrials.gov, the World Health Organization International Clinical Trials Registry Platform, and hand-searching reference lists of included studies and relevant reviews. The search combined Medical Subject Headings (MeSH) and free-text terms for “ranolazine.” We did not include additional search terms to maximize search sensitivity. No language or date restrictions were applied.

Study Selection

All identified records were imported into Rayyan (Rayyan Systems Inc., Cambridge, MA) for deduplication and initial screening [7]. Two authors independently reviewed titles and abstracts and subsequently assessed the full texts of potentially relevant articles. Differences in judgment were resolved by discussion, and a third reviewer was consulted when consensus could not be reached. Studies providing quantitative outcome data were included in the meta-analysis, while those meeting inclusion criteria but lacking extractable data were incorporated into the narrative synthesis.

Data Extraction

Data were independently extracted by two reviewers using a pre-piloted standardized form. Extracted information included study characteristics (author, the year of publication, country, study design {RCT, non-randomized trial, and other observational designs}, setting, sample size in intervention and control groups, population type, and the inclusion of special subgroups such as patients with diabetes or elderly patients). Participant characteristics, including mean age, sex distribution, comorbidities, a history of prior revascularization (PCI or CABG), and ranolazine dosage, were also collected. Outcome data included measures related to PCI requirements, angina symptom scores, QoL scores, exercise tolerance, and other relevant endpoints. Outcomes were recorded as reported in the original studies, as either means or frequencies, along with any covariates used for adjustment. A third reviewer audited the extracted data for consistency.

Risk of Bias Assessment

Two reviewers independently evaluated the risk of bias in the included studies. For RCTs, assessment was carried out using the Cochrane Risk of Bias 2 (RoB 2) tool, which examines potential bias related to randomization, deviations from intended interventions, incomplete outcome data, outcome measurement, and selective reporting [8]. Non-randomized interventional studies were appraised with the ROBINS-I tool, which considers confounding, participant selection, intervention classification, deviations from intended interventions, missing data, outcome assessment, and reporting bias [9]. Any disagreements were resolved by consensus, with a third reviewer consulted when necessary. Each study was subsequently rated as low, moderate, or high risk of bias according to predefined criteria.

Statistical Analysis

Random-effects meta-analyses were conducted using the “meta” package in R version 4.4.3 [10]. The restricted maximum likelihood estimator method was used to estimate the between-study variance: τ². For studies with multiple follow-up assessments, the latest follow-up data were used. Continuous outcomes such as SAQ scores in different domains (angina frequency, angina stability, physical limitation, QoL, and treatment satisfaction), exercise duration, time to angina onset, time to 1-mm ST depression, and angina frequency were pooled as mean differences (MDs) with 95% confidence intervals (CIs). Dichotomous outcomes such as the incidence of MI, revascularization (PCI and CABG), and hospitalization were summarized using risk ratios (RRs) with 95% CIs. Heterogeneity across studies was assessed using the I² statistic, with I² values greater than 75% considered indicative of substantial heterogeneity [11]. Forest plots were generated for all pooled estimates to assess effect sizes and CIs visually [12]. Subgroup analyses were conducted based on the follow-up duration of more than six months and on disease subtypes (obstructive or microvascular) to evaluate the effects of ranolazine. Sensitivity analysis was performed by omitting studies with a high risk of bias.

Publication Bias and Certainty of Evidence

Potential publication bias was explored through the visual examination of funnel plots and confirmed with Egger’s regression test, using a significance threshold of p< 0.10 wherever the number of studies available was more than 10. The certainty of the evidence was assessed using the Grading of Recommendations Assessment, Development, and Evaluation (GRADE) methodology, and a summary of findings table was generated with GRADEpro Guideline Development Tool (GDT) (Evidence Prime, Hamilton, Canada; 2023, available from gradepro.org).

Results

Literature Search

Our search strategy identified 6033 unique records, including 1227 from PubMed, 1252 from EMBASE, 1702 from Web of Science, 190 from the Cochrane Central Register of Controlled Trials, and 1662 from Scopus. After removing 3161 duplicate entries, 2872 records remained for title and abstract screening. Of these, 2743 were excluded because they did not meet the predefined eligibility criteria related to the study population, intervention, comparator, or outcomes. The full texts of the remaining 129 articles were sought for retrieval and detailed assessment. Of these, 115 were finally retrieved, while 14 were not retrieved as they did not match the specified publication type. Upon full-text review, 46 studies were excluded: 11 were conference abstracts of the main study, six did not evaluate ranolazine, eight were review articles, six did not match the publication type, six assessed different outcomes, four did not match the population in question, four were of varying study designs, and one was a duplicate entry. Ultimately, 69 studies met all inclusion criteria and were included in the final synthesis. Among these, data from 40 studies were used for quantitative meta-analysis of different outcomes (Figure 1).

**Figure 1:**
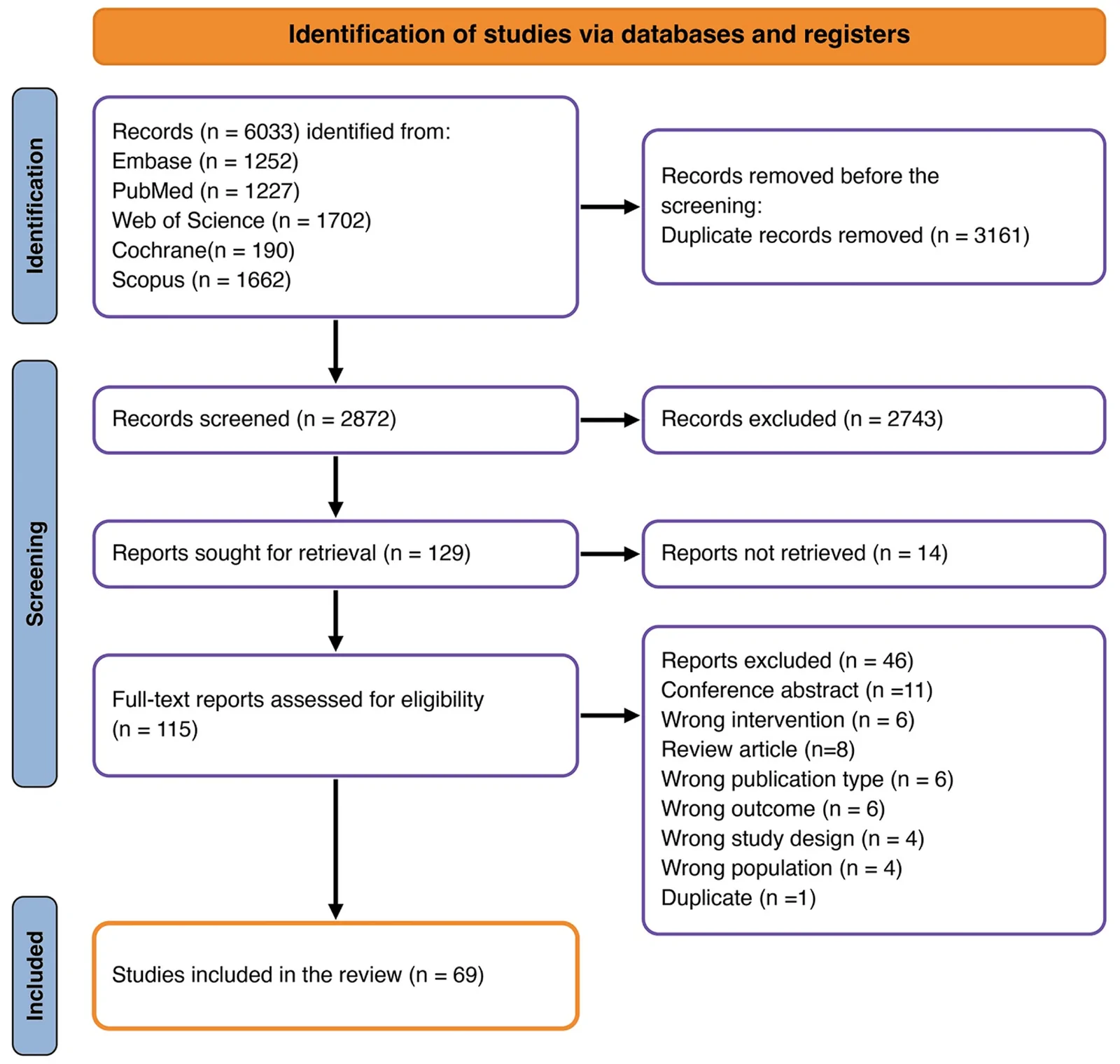
Flowchart depicting article selection and screening process

Characteristics of the Included Studies

The included studies assessing the efficacy and safety of ranolazine in patients with various forms of angina and ischemic heart conditions were diverse in design, population, and setting. Among them, a mix of RCTs, non-randomized controlled trials, cohort studies, prospective observational studies, retrospective analyses, and case series was identified. Geographically, most studies were conducted in the USA and Europe, with additional contributions from countries such as Brazil, India, Pakistan, Canada, and Russia. Sample sizes varied widely, ranging from as few as seven participants to over 19000 in large database cohort analyses. The participant populations predominantly included patients with CSA, microvascular angina, non-obstructive CAD, coronary microvascular dysfunction, or refractory angina, often on background antianginal therapy and frequently having undergone prior revascularization procedures such as PCI or CABG. The average participant age ranged from 50 to 75 years, with most studies reporting a higher proportion of male participants; however, several recent trials focused specifically on female populations or those with gender-balanced recruitment. Special groups included patients with diabetes mellitus, those with incomplete revascularization, and those with documented coronary flow reserve (CFR) abnormalities. The characteristics of the included studies are summarized in Table 1.

**Table 1:** Comprehensive overview of the included studies ACS, acute coronary syndrome; AE, adverse event; ANOCA, angina with non-obstructive coronary arteries; BB, beta-blocker; BID, twice per day; BP, blood pressure; CABG, coronary artery bypass grafting; CAD, coronary artery disease; CAN, coronary artery narrowing; CCB, calcium channel blocker; CCS, Canadian Cardiovascular Society; CFR, coronary flow reserve; CHD, coronary heart disease; CMD, coronary microvascular dysfunction; CMRI, cardiac magnetic resonance imaging; CMR, cardiac magnetic resonance; CMVD, coronary microvascular disease; COAD, coronary obstructive artery disease; CRT, cardiac resynchronization therapy; DLP, dyslipidemia; DM, diabetes mellitus; DT, deceleration time; E/A, early-to-late diastolic transmitral flow velocity ratio; ETT, exercise tolerance test; GTN, glyceryl trinitrate; HbA1c, glycosylated hemoglobin; HR, heart rate; ICR, incomplete coronary revascularization; IHD, ischemic heart disease; IVRT, isovolumic relaxation time; LVEF, left ventricle ejection fraction; MACE, major adverse cardiovascular events; MetS, metabolic syndrome; MI, myocardial infarction; MPI, myocardial perfusion imaging; MPRI, myocardial perfusion reserve index; MVPA, moderate-to-vigorous physical activity; NA, not available; NOCAD, non-obstructive coronary artery disease; NR, not reported; OD, once daily; PCI, percutaneous coronary intervention; QoL, quality of life; QTc, corrected QT; RCT, randomized controlled trial; RDS, Rose Dyspnea Scale; RFa, radiofrequency ablation; RPP, rate–pressure product; SAQ, Seattle Angina Questionnaire; SCAD, stable coronary artery disease; SF-36, 36-Item Short Form Survey; SIHD, stable ischemic heart disease; SLN, sublingual nitroglycerin; SPECT, single-photon emission computed tomography; SR, symptom relief; T2DM, type 2 diabetes mellitus; TID, three times a day; TMT, treadmill test; Tx, treatment; 6MWD, six-minute walk distance

Author year	Design	Country	Participants (intervention)	Participants (control)	Population	Mean age	Gender (% male)	Dose of ranolazine	Follow-up duration	Outcomes
Storey et al., 2020 [13]	Prospective registry	USA	158	158	Refractory angina, high cardiac risk	64	73%	500 mg BID, titrated to 1000 mg BID	3 years	SR
Alexopoulos et al., 2016 [14]	Cohort	The Netherlands	189	189	Chronic stable angina, 56.6% prior revascularization	71.5	70.4%	375 mg BID, >500/750 mg BID	6 months	CCS, SLN use
Dourado et al., 2022 [15]	Retrospective, pre-post	Brazil	9	10	Refractory angina (CCS 3/4), ≥3 antianginals	61	NA	500 mg BID	3 months	CCS, QTc
Villano et al., 2013 [16]	RCT	USA	15	15	Stable microvascular angina, CFR < 2.5	NA	NA	375 mg BID	4 weeks	Angina frequency, stability, physical limitation, QoL, Tx satisfaction, exercise, ST depression, RPP
Willis et al., 2019 [17]	RCT	USA	13	16	CCS	68	CCS	500 mg BID × one week >1000 mg BID	14 weeks (two-week titration + 12-week exercise training)	Angina frequency, angina stability, physical limitation, quality of life, treatment satisfaction
Alexander et al., 2016 [18]	RCT	USA	1207	1182	Chronic angina, post-PCI ICR	63	80%	1000 mg BID	12 months; median of 643 days	Angina frequency, stability, physical limitation, QoL, Tx satisfaction
Page et al., 2015 [19]	Cohort	USA	2002	2002	Chronic stable angina, some with DM	66	63%	NR	12 months	SR
Stone et al., 2010 [20]	RCT	USA	170	170	Stable ischemic heart disease, exertional angina	≥21	NR	500, 1000, 1500 mg BID (one week each)	4 weeks	SR
Chaitman et al., 2004 [21]	RCT	USA	191	191	Chronic severe stable angina	64.3	73.3%	500, 1000, 1500 mg BID (one week each)	1 week/dose; 2-year open-label	Exercise, angina, ST depression
Rambarat et al., 2019 [22]	RCT	The Netherlands	81	81	Symptomatic, non-obstructive CAD, CFR < 2.5	55	1.2%	500 mg BID (one week), then 1000 mg BID	6 weeks (two weeks, ranolazine; two-week washout; two weeks, placebo)	SR
Sinha et al., 2024 [23]	RCT	USA	40	47	ANOCA, some CMD	61	38%	375 mg BID (one week), then 500/750 mg BID	9 weeks (four weeks, ranolazine; one-week washout; four weeks, amlodipine)	SR
Borzi et al., 2018 [24]	Prospective, pre-post	USA	94	94	SCAD (73% COAD; 27% NOCAD), symptomatic despite BB/CCB	69	68%	375-500 mg BID	6 months	Angina frequency, stability, physical limitation, QoL, Tx satisfaction
Tolunay, 2021 [25]	Non-RCT	England	30	30	CAD with angina	68.35	55%	750 mg BID, then 1000 mg/day	1 month	SR
Rayner-Hartley et al., 2020 [26]	Retrospective, pre-post	USA	31	31	Microvascular angina, severe refractory, no obstructive CAD	57	23%	500-2000 mg daily	~6 weeks (median of 17 months)	SR
Koh et al., 2020 [27]	RCT	USA	11	11	Angina, NOCAD, 54.5% CFR < 2.0	NR	NR	500 mg BID (two weeks), then 1000 mg BID	12 weeks	Angina frequency, stability, physical limitation, QoL, Tx satisfaction, CFR
Caminiti et al., 2016 [28]	RCT	The Netherlands	20	20	Overweight/obese nondiabetic CHD, insulin resistance	62.4	77.5%	500 mg BID	12 weeks	SR
Mehta et al., 2019 [29]	Open-label, pre-post	USA	159	159	Women with IHD, ≥2 angina episodes/week	65	0%	500 mg BID	4 weeks	SR
Ahmed et al., 2017 [30]	Open-label, pre-post	USA	7	7	Type 1 CMVD, non-obstructive CAD, HTN, DLP	57.6	57%	500 mg BID (seven days), then 1000 mg BID	4 weeks	CFR
Safdar et al., 2017 [31]	RCT	USA	21	10	Chest pain, CMD	50	29%	500 mg BID (one week), then 1000 mg BID	30 days	CFR
Zweiker et al., 2019 [32]	Prospective cohort	Austria	289	292	Stable angina, 57.2% prior revascularization	73.6	55.5%	375 mg BID, titrated to 500/750 mg BID	12 weeks	Angina frequency, SLN use
Bress et al., 2018 [33]	Retrospective cohort	USA	4770	32290	Veterans with chronic stable angina	67	98%	NR	Median of 3.4 years	MI, PCI, CABG
Shah et al., 2017 [34]	RCT	England	21	19	Diabetic, non-obstructive CAD, 43% prior revascularization	64	51%	500 mg BID (one week), then 1000 mg BID	8 weeks (four weeks, ranolazine; three-day washout; four weeks, placebo)	SR
Phelps et al., 2012 [35]	Cohort	USA	881	3664	Angina, changed antianginal therapy	61	NR	NR	12 months	PCI, CABG, revascularization, hospitalization, SLN
Weisz et al., 2016 [36]	RCT	England	1332	1319	Chronic angina, post-PCI ICR	63.4	79%	500 mg BID (seven days), then 1000 mg BID	Median of 643 days	MI, hospitalization, cardiac death, AE, mortality, any revascularization
Tagliamonte et al., 2015 [37]	RCT	USA	29	29	Angina, non-obstructive CAD	66	67%	375 mg BID (four weeks), then 500 mg BID	8 weeks	Angina frequency, stability, physical limitation, QoL, Tx satisfaction, ECG frequency
Meyer et al., 2019 [38]	Retrospective cohort	USA	19574	89167	SIHD	67	59%	NR	12 months	MACE any revascularization
Kanorskii and Smolenskaya, 2016 [39]	RCT	Russia	53	54	Coronary heart disease and functional class II/III stable angina	NR	NR	500 mg BID	6 months	SR
Gutierrez et al., 2015 [40]	RCT	USA	1317	1334	Chronic angina with non-ST-elevation acute coronary syndrome post-PCI	NR	NR	IV 200 mg bolus, 80 mg/hour (12-96 hours), then 1000 mg BID oral	12 months	MACE, MI, cardiac death, hospitalization, any revascularization
Greene et al., 2012 [41]	Case-series	USA	18	18	Men with ≥3 angina episodes/week despite max therapy	66	100%	500 or 1000 mg BID	2-12 weeks	ECG for angina weekly, QTc
Mehta et al., 2011 [42]	RCT	USA	20	20	Women with ischemia, no obstructive CAD, abnormal CMR	65	0%	500 mg BID (two weeks), then 1000 mg BID	10 weeks (four-week treatment; two-week washout)	Angina frequency, stability, physical limitation, QoL, Tx satisfaction
Muhlestein and Grehan, 2013 [43]	Cross-sectional, pre-post	New Zealand	1785	1775	Chronic angina, no prior revascularization	64	36	NR	Not applicable (recall-based)	SR
Bennett et al., 2014 [44]	Cohort	USA	100	43	Refractory angina clinic patients	64.1	75	500 mg BID, titrated to 1000 mg BID	12 months	SR
Wilson et al., 2009 [45]	RCT	USA	1789	1776	Chronic angina with ACS (MERLIN-TIMI 36)	NR	NR	IV for 12-96 hours, then 1000 mg BID oral	Median of 350 days	ECG exercise duration, MACE overall, MACE all mortality, time 1-mm ST depression
Deshmukh et al., 2009 [46]	RCT	England	27	27	CAD, post-CABG/PCI, CCS class I angina	64	70	500 mg BID (one week), then 1000 mg BID	12 weeks (six weeks per treatment)	SR
Karwatowska-Prokopczuk et al., 2013 [47]	RCT	England	6492	3256	Non-ST-elevation acute coronary syndrome at moderate-to-high risk for recurrent ischemic events	63	NR	IV, then 1000 mg BID oral; 500 mg if renal impairment	Median of 348 days	SR
Reeder et al., 2012 [48]	Cohort	USA	35	35	Veterans with chronic stable angina	67.8	100%	500 mg BID, titrated to 1000 mg BID	3 months	SAQ (angina frequency, physical limitation, Tx satisfaction, QoL, stability), adverse events, cardiac death
Olympios et al., 2024 [49]	Non-RCT	Switzerland	1101	1101	Adults with angina, recently started on ranolazine	71.3	73.29%	375 mg BID	3 months	SLN use, angina frequency, CCS, time to angina, QoL, discontinuation
Khot et al., 2017 [50]	RCT	India	72	72	Chronic stable angina, ≥2-3 episodes/week	58.74	72.2%	500 mg BID or 1 g OD	6 months	Angina frequency, exercise (TMT), ECG, SAQ, stress ECG
Shammas et al., 2015 [51]	RCT	New Zealand	24	25	Ischemic cardiomyopathy, angina/dyspnea, LVEF ≤ 40%	71.5	82.1%	500 mg BID (one week), then 1000 mg BID	16 weeks	SAQ, dyspnea (RDS)
Lee et al., 2025 [52]	RCT	Singapore	38	35	CAD, stable angina ≥ 3 months	60.4	100	500-1000 mg BID	12 weeks	Time to angina, ST depression, SLN use, angina frequency, ETT, laboratory parameters, AEs
Calcagno et al., 2020 [53]	RCT	Switzerland	49	49	CAD, post-PCI, CCS II/III angina	62.6	89.8%	375 mg BID	30 days	Exercise, time to angina, RPP
Rahmani et al., 2022 [54]	RCT	Canada	31	31	Post-stent, persistent angina	58.81	59.68%	1000 mg daily	4 weeks	Exercise, chest pain severity
Murray and Colombo, 2016 [55]	Cohort	USA	51	54	Chronic disease patients	64.51	74.29%	500-1000 mg BID	6.19 years	Cardiac death, MI, PCI, CABG, revascularization, AEs, ACS, MPI, RFa, CAN
Birkeland et al., 2017 [56]	RCT	Canada	16	24	Ischemia, no obstructive CAD, CMD	54.3	3%	500 mg BID (one week), 1000 mg BID (three weeks)	2 weeks	SAQ (angina frequency, physical limitation, Tx satisfaction, QoL, stability), RPP, MPRI
Chaturvedi et al., 2013 [57]	RCT	India	30	31	Chronic stable angina, 18-60 years	60.3	88.3%	500 mg BID	8 weeks	Angina frequency, AEs, BP, HR, hematological, biochemical
Khalil-ur-Rehman et al., 2017 [58]	Non-RCT	Pakistan	53	53	Angina, increased chest pain frequency	NR	100%	750 mg BID	6 weeks	Angina frequency, SLN use, pain duration, hemodynamics, ECG, laboratory parameters, AEs
Jain et al., 1990 [59]	RCT	England	14	14	Stable effort-induced angina, 3-10-minute exercise	59.9	78.57%	30 mg TID, then 60 mg TID	6 weeks	Exercise, time to angina, ST depression, RPP, angina frequency, GTN use, AEs
Cocco et al., 1992 [60]	RCT	Switzerland, Belgium	52	52	Chronic stable angina, symptomatic despite BB/diltiazem	NR	NR	10, 60, 120, 240 mg	NR	Exercise, time to angina, ST depression, plasma levels, hemodynamics
Singh et al., 2014 [61]	RCT	England	61	61	Stable CAD	62	69%	500 mg BID	6 months	ETT, HbA1c, angina scores, lipids, BMI, AEs
Sendón et al., 2012 [62]	RCT	Spain	174	84	CAD, chronic exertional angina, max therapy	65.3	82.6%	750 or 1000 mg BID	12 weeks	Exercise, time to angina, ST depression, RPP, AEs
Thadani et al., 1994 [63]	RCT	USA	240	79	Chronic stable angina, ECG ischemia	65.26	79%	30, 60, 120 mg TID	4 weeks	Exercise, time to angina, ST depression, RPP, GTN use, angina episodes
Kosiborod et al., 2013 [64]	RCT	USA, Israel	462	465	T2DM, CAD, symptomatic angina	64	61%	500 mg BID (one week), then 1000 mg BID	12 weeks	MI, AEs, SLN use, angina frequency, mortality
Stone et al., 2006 [65]	RCT	USA, Russia	281	284	CAD, ≥3 angina episodes/week despite amlodipine	61.65	72.5%	500 mg (one week), then 1000 mg BID	6 weeks	SLN use, angina frequency, MI, AEs, SAQ, hemodynamics, ECG
Rousseau et al., 2005 [66]	RCT	USA, Belgium, Switzerland	158	158	Chronic angina pectoris	59	89%	400 mg TID	1 week	AEs, time to angina, exercise, ST depression, hemodynamics
Morrow et al., 2007 [67]	RCT	Multiple	3279	3281	Non-ST-elevation ACS	64	65.1%	1000 mg BID after IV loading	Median of 348 days	SAQ-angina frequency, cardiac death, MI, revascularization, mortality, hospitalization, AEs
Koren et al., 2007 [68]	RCT	USA	746	746	Severe chronic stable angina	<65	77.7%	500, 750, 1000 mg BID	Mean of 2.82 years	Safety, ranolazine intolerance, mortality
Venkataraman et al., 2009 [69]	NR	USA	21	21	CAD, ischemia on SPECT MPI	65	67%	1000 mg BID	4 weeks	Exercise, MPI, perfusion defects, angina, QT, LVEF
Patel et al., 2015 [70]	RCT	England	50	50	Refractory angina despite revascularization, therapy	65.2	87%	375 mg BID	1 month	CCS, SLN use, AEs, QTc, 6MWD, GTN use
Pylova and Tseluyko, 2022 [71]	RCT	Ukraine	16	37	Ischemia, no obstructive CAD	57	33.9%	1000 mg BID	6 months	Exercise, RPP, ventricular arrhythmias
Kuchmin et al., 2018 [72]	RCT	USA, Russia	21	21	SIHD with diastolic dysfunction			1000 mg BID	4 weeks	Diastolic function, E/A ratio, DT, IVRT
Ling et al., 2013 [73]	Cohort	USA	150	150	Refractory angina resistant to therapy/revascularization	66	64%	860 ± 278 mg/day to 1616 ± 526 mg/day	12 months	MI, PCI, CABG, hospitalizations, CCS, AEs
Malhotra et al., 2019 [74]	RCT	USA	11	16	SIHD, angina, deferred PCI (FFR ≥ 0.81)	NR	NR	1000 mg BID	NR	SLN use, physical activity, MVPA, steps/day, SIHD guidelines
Coleman et al., 2018 [75]	RCT	USA	457	461	T2DM, chronic stable angina	NR	NR	500 mg BID (one week), then 1000 mg BID	8 weeks	SF-36 physical/mental health, domain scores
Bavry et al., 2017 [76]	RCT	USA	25	25	Chronic stable angina, myocardial ischemia	67	NR	500 mg BID (one week), then 1000 mg BID	16 weeks	SAQ (angina frequency, physical limitation, Tx satisfaction, QoL, stability), ischemia-driven events
Bairey Merz et al., 2016 [77]	RCT	USA	70	72	Ischemia, no obstructive CAD, abnormal CRT/CMRI	55.2	4%	500 mg BID (one week), then 1000 mg BID	2 weeks	SAQ (angina frequency, physical limitation, Tx satisfaction, QoL, stability), angina frequency, SLN use, MPRI
Egea-Serrano et al., 2018 [78]	RCT	Spain	7	7	Angina, non-obstructive CAD	65	57.14%	375 mg BID	3 months	CFR, functional capacity, MetS, safety
Timmis et al., 2006 [79]	RCT	UK	554	269	Diabetic, chronic stable angina (CARISA subset)	64.22	77.4%	750 and 1000 mg BID	2 years	Exercise, time to angina, ST depression, SLN use, angina frequency, HbA1c, glucose, lipids
Chaitman et al., 2004 [80]	RCT	USA	554	269	Chronic stable angina, symptomatic	63.97	77.5%	750 and 1000 mg BID	12 weeks	Exercise, time to angina, ST depression, ECG ischemia, safety
Pepine and Wolff, 1999 [81]	RCT	USA	159	159	Chronic stable angina, responsive to therapy	64.2	72%	267 mg TID, 400 mg BID/TID	5 weeks	Time to angina, exercise, ST depression, plasma levels, hemodynamics, safety

Risk of Bias Across Included Studies

Randomized studies: Risk of bias in RCTs was assessed with the RoB 2 tool across five domains (Figure 2). Overall, most trials were judged to have a low-to-moderate risk of bias. The primary concern was the randomization process, particularly the limited details provided on sequence generation and allocation concealment. In contrast, deviations from intended interventions and selective reporting were typically considered low risk. Missing outcome data and outcome measurement presented greater variability, with several studies rated at high risk, reflecting incomplete follow-up and potential detection bias. Overall, while several studies were assessed as low risk, a considerable proportion raised some concerns, particularly related to randomization procedures and outcome measurement. Nevertheless, the consistency of effect estimates across trials suggests that these risks are unlikely to have materially influenced the pooled findings.

**Figure 2:**
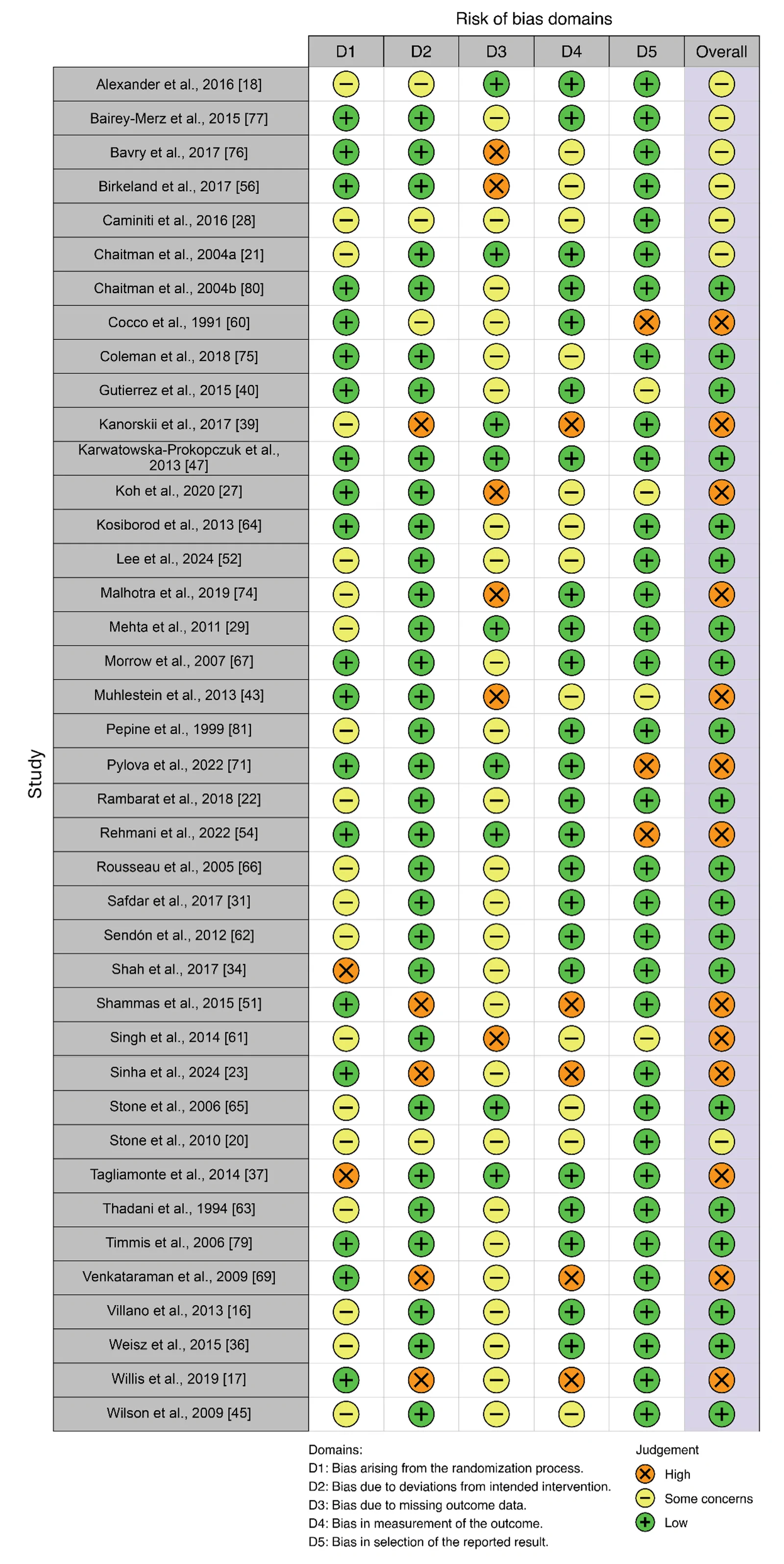
RoB 2 for the risk of bias assessment of RCTs RoB 2, Risk of Bias 2; RCTs, randomized controlled trials

Non-randomized studies: Risk of bias was assessed using the ROBINS-I tool across seven domains (Figure 3). The most frequent concerns were related to confounding and classification of interventions, with nearly all studies rated as being at critical risk, reflecting the inherent limitations of observational designs and the lack of adequate adjustment for potential confounders. Deviations from intended interventions and outcome measurement also frequently showed moderate-to-critical risk, particularly in studies lacking blinding or standardized assessment methods. By contrast, participant selection and selective reporting were more often judged to be at low risk, suggesting that these issues were less problematic. Missing outcome data varied across studies, with some judged at low risk but others at moderate risk due to incomplete follow-up.

**Figure 3:**
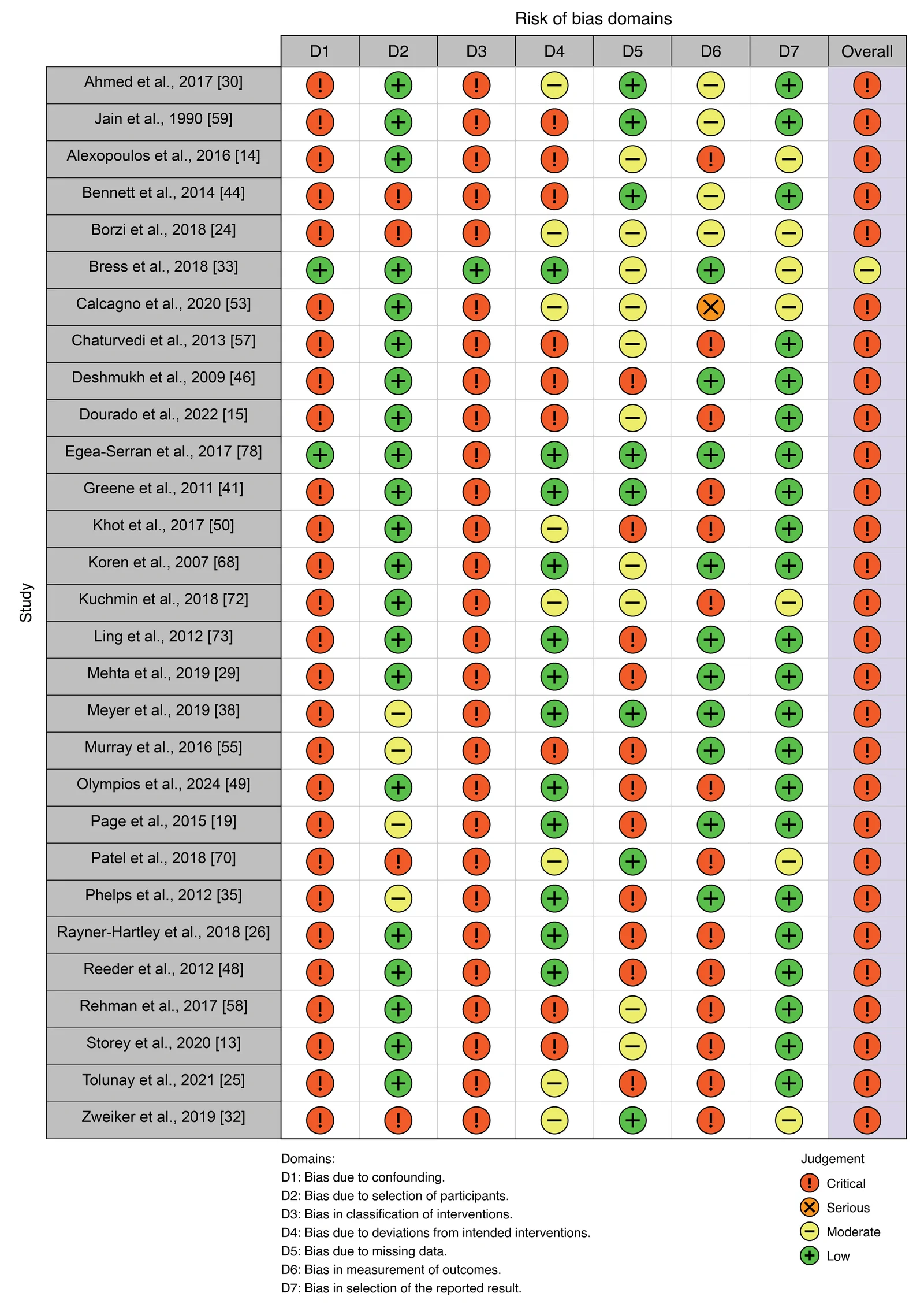
ROBINS-I risk of bias assessment of non-RCTs RCTs: randomized controlled trials

SAQ Domains

The analysis showed statistically significant improvements in both the angina frequency and angina stability domains (Table 2).

**Table 2:** Primary meta-analysis of Seattle Angina Questionnaire domains and exercise parameters CI, confidence interval; QoL, quality of life; NA, not available

	Overall		>6 months		Microvascular disease		Obstructive disease	
Outcome	Number of studies	Pooled estimate with CI	Number of studies	Pooled estimate with CI	Number of studies	Pooled estimate with CI	Number of studies	Pooled estimate with CI
Seattle Angina Questionnaire: domains								
Angina frequency	9	4.63 (1.06-8.21)	2	1.08 (-1.08-3.23)	4	7.89 (0.94-14.84)	5	2.23 (-0.14-4.60)
Angina stability	7	12.00 (1.65-22.36)	1	NA	4	20.35 (9.92-30.78)	3	0.22 (-2.31-2.74)
Physical limitation	7	2.94 (-4.05-9.92)	1	NA	4	6.29 (2.59-9.99)	3	-4.14 (-18.94-10.66)
QoL	7	6.16 (-0.46-12.77)	1	NA	4	10.85 (2.80-18.91)	3	-0.18 (-2.13-1.77)
Treatment satisfaction	7	0.01 (-5.22-5.25)	1	NA	4	1.05 (-8.45-10.56)	3	-0.32 (-1.68-1.05)
Exercise parameters								
Exercise duration	11	33.15 (27.39-38.92)	3	31.40 (26.59-36.22)	2	39.98 (-22.17-102.13)	9	33.10 (27.27-38.94)
Time to angina	9	42.87 (34.23-51.51)	3	48.91 (44.40-53.43)	NA	NA	9	42.87 (34.23-51.51)
Time to 1-mm ST depression	10	43.23 (33.81-52.65)	3	45.94 (40.39-51.49)	1	NA	9	42.58 (33.10-52.05)

Based on nine studies, there was a mean improvement of 4.63 points (95% CI: 1.06-8.21) in the angina frequency domain and 12.00 points (95% CI: 1.65-22.36) in the angina stability domain. No statistically significant effects were observed for physical limitation (MD, 2.94; 95% CI, -4.05-9.92), QoL (MD, 6.16; 95% CI, -0.46-12.77), or treatment satisfaction (MD, 0.01; 95% CI, -5.22-5.25) domains (Figure 4a-4e).

**Figure 4:**
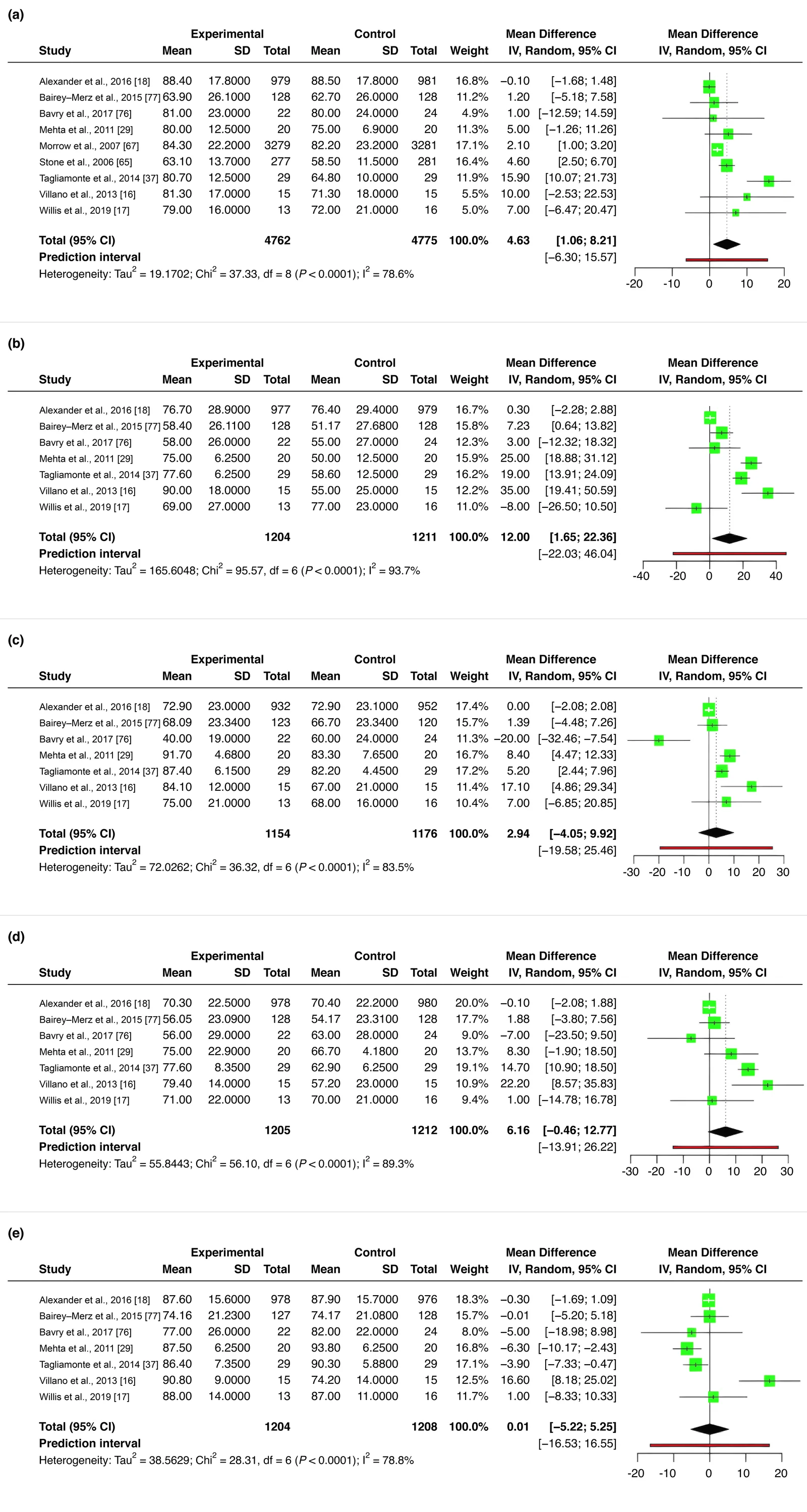
Forest plot of the effect of ranolazine on (a) SAQ angina frequency, (b) SAQ angina stability, (c) SAQ physical limitation, (d) SAQ quality of life, and (e) SAQ treatment satisfaction SAQ, Seattle Angina Questionnaire; SD, standard deviation; CI, confidence interval; df, degrees of freedom; IV, inverse variance

When subgroup analysis was performed on studies with a follow-up of >6 months, the improvement in angina frequency was no longer statistically significant (MD, 1.08; 95% CI, -1.08-3.23). There was insufficient data to produce pooled estimates for the other SAQ domains in this subgroup. In the subgroup analysis of patients with microvascular disease, the benefits were more pronounced and widespread. Statistically significant improvements were seen not only in angina frequency (MD, 7.89; 95% CI, 0.94-14.84) and angina stability (MD, 20.35; 95% CI, 9.92-30.78) but also in physical limitation (MD, 6.29; 95% CI, 2.59-9.99) and QoL (MD, 10.85; 95% CI, 2.80-18.91); treatment satisfaction did not show a significant improvement in this subgroup (MD, 1.05; 95% CI, -8.45-10.56) (Figure 5a-5e).

**Figure 5:**
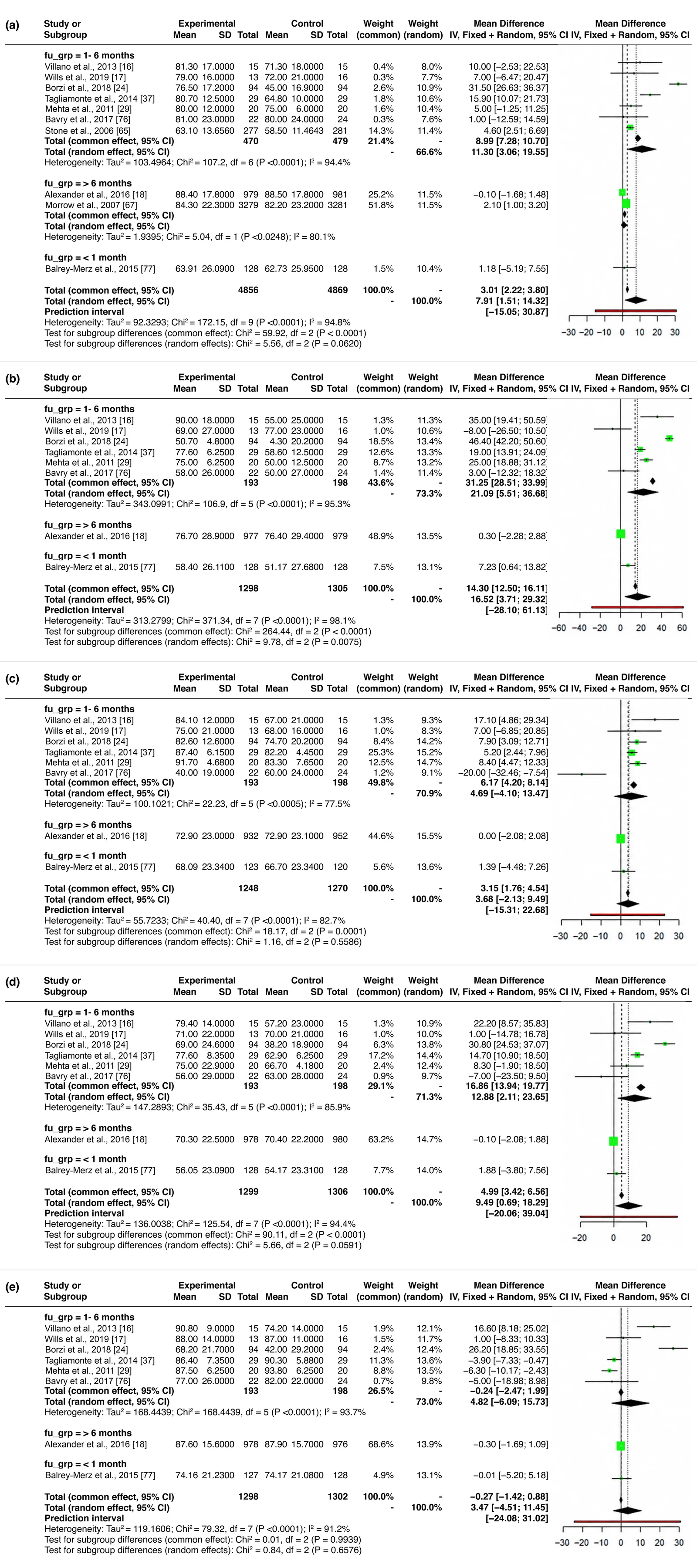
Forest plot of the effect of ranolazine based on follow-up duration on (a) SAQ angina frequency, (b) SAQ angina stability, (c) SAQ physical limitation, (d) SAQ quality of life, and (e) SAQ treatment satisfaction SAQ, Seattle Angina Questionnaire; SD, standard deviation; CI, confidence interval; df, degrees of freedom; IV, inverse variance

Conversely, in the subgroup of patients with obstructive disease, no statistically significant improvements were found across any of the five SAQ domains.

Exercise Parameters

The overall analysis demonstrated significant improvements in objective exercise capacity. Pooled data from 11 studies showed increases in exercise duration (MD, 33.15 seconds; 95% CI, 27.39-38.92), time to angina onset (MD, 42.87 seconds; 95% CI, 34.23-51.51), and time to 1-mm ST depression (MD, 43.23 seconds; 95% CI, 33.81-52.65). These benefits were maintained in studies with >6 months of follow-up and in the obstructive disease subgroup. However, in the microvascular disease subgroup, the improvement in exercise duration was not statistically significant (MD, 39.98 seconds; 95% CI, -22.17-102.13), with no pooled data available for other exercise parameters.

Major Adverse Cardiac Events and Adverse Events

The overall analysis found a statistically significant reduction in the risk of any MACE (pooled estimate: RR, 0.89; 95% CI, 0.84-0.94), CABG (RR, 0.55; 95% CI, 0.35-0.86), any revascularization such as PCI or CABG (RR, 0.77; 95% CI, 0.64-0.91), MI (RR, 0.91; 95% CI, 0.84-0.98), and hospitalization (RR, 0.87; 95% CI, 0.77-0.98) (Table 3).

**Table 3:** Primary meta-analysis of cardiovascular outcomes CI, confidence interval; MACE, major adverse cardiovascular events; MI, myocardial infarction; NA, not available; PCI, percutaneous coronary intervention; CABG, coronary artery bypass grafting

	Overall		>6 months		Microvascular disease		Obstructive disease	
Outcome	Number of studies	Pooled estimate with CI	Number of studies	Pooled estimate with CI	Number of studies	Pooled estimate with CI	Number of studies	Pooled estimate with CI
MACE								
Any MACE	5	0.89 (0.84-0.94)	5	0.89 (0.84-0.94)	NA	NA	5	0.89 (0.84-0.94)
PCI	3	0.80 (0.55-1.18)	2	0.98 (0.71-1.36)	NA	NA	1	NA
CABG	3	0.55 (0.35-0.86)	2	1.05 (0.18-6.25)	NA	NA	1	NA
Any revascularization	7	0.77 (0.64-0.91)	6	0.83 (0.75-0.93)	NA	NA	5	0.83 (0.68-1.00)
MI	8	0.91 (0.84-0.98)	6	0.91 (0.84-0.98)	1	NA	7	0.94 (0.84-1.06)
Hospitalization	5	0.87 (0.77-0.98)	4	0.88 (0.75-1.03)	1	NA	4	0.88 (0.75-1.03)
Cardiac death	5	1.02 (0.86-1.20)	5	1.02 (0.86-1.20)	NA	NA	5	1.02 (0.86-1.20)
All mortality	4	1.00 (0.86-1.16)	3	1.00 (0.86-1.16)	NA	NA	4	1.00 (0.86-1.16)
Adverse events	11	1.45 (1.27-1.66)	3	1.58 (1.27-1.97)	NA	NA	11	1.45 (1.27-1.66)

There was no significant effect on PCI, cardiac death, or all-cause mortality. However, the risk of adverse events significantly increased (RR, 1.45; 95% CI, 1.27-1.66).

In the >6-month follow-up subgroup, the significant risk reductions for any MACE (RR, 0.89; 95% CI, 0.84-0.94), any revascularization such as PCI or CABG (RR, 0.83; 95% CI, 0.75-0.93), and MI (RR, 0.91; 95% CI, 0.85-0.99) were maintained. However, the benefits of CABG and hospitalization were no longer statistically significant in this longer-term follow-up. The increased risk of adverse events remained significant (RR, 1.58; 95% CI, 1.27-1.97). No data were available on MACE or adverse CABG events in the microvascular disease subgroup. In the obstructive disease subgroup, the only MACE outcome that remained statistically significant was the reduction in any MACE (RR, 0.89; 95% CI, 0.84-0.94). The risk reductions for any revascularization, such as PCI or CABG, MI, and hospitalization, were no longer statistically significant in this subgroup. The increased risk of adverse events remained significant and was identical to the overall analysis (RR, 1.45; 95% CI, 1.27-1.66).

Sensitivity Analysis: Results Excluding High-Risk Studies

A sensitivity analysis was performed, excluding high-risk studies, to evaluate the robustness of the primary findings.

SAQ domains: After excluding high-risk studies, the improvement in angina frequency remained statistically significant but was attenuated (2.48; 95% CI, 0.39-4.56). Angina’s stability also remained significant (15.85; 95% CI, 0.71-30.98) (Table 2).

Exercise parameters: The improvements in exercise capacity were highly robust. Significant gains were maintained for exercise duration (32.08 seconds; 95% CI, 26.84-37.32), time to angina onset (42.87 seconds; 95% CI, 34.23-51.51), and time to 1-mm ST depression (43.23 seconds; 95% CI, 33.81-52.65). The results for time to angina onset and time to 1-mm ST depression were identical to the primary analysis. In contrast, for exercise duration, only one study was excluded due to a high risk of bias (Table 4).

**Table 4:** Sensitivity analysis excluding studies with high risk of bias: clinical endpoints CI, confidence interval; QoL, quality of life

	Overall		>6 months		Microvascular disease		Obstructive disease	
Outcome	Number of studies	Pooled estimate with CI	Number of studies	Pooled estimate with CI	Number of studies	Pooled estimate with CI	Number of studies	Pooled estimate with CI
Seattle Angina Questionnaire: domains								
Angina frequency	6	2.48 (0.39-4.56)	2	1.08 (-1.08-3.23)	3	3.91 (-0.30-8.12)	3	2.12 (-0.43-4.68)
Angina stability	4	15.85 (0.71-30.98)	1	NA	3	21.39 (5.79-36.98)	1	NA
Physical limitation	4	5.25 (-0.99-11.48)	1	NA	3	7.64 (0.16-15.11)	1	NA
QoL	4	6.25 (-2.22-14.72)	1	NA	3	9.45 (-1.69-20.60)	1	NA
Treatment satisfaction	4	1.93 (-7.00-10.86)	1	NA	3	2.99 (-10.07-16.05)	1	NA
Exercise parameters								
Exercise duration	9	32.08 (26.84-37.32)	3	31.40 (26.59-36.22)	1	NA	8	32.03 (26.76-37.29)
Time to angina	9	42.87 (34.23-51.51)	3	48.91 (44.40-53.43)	NA	NA	9	42.87 (34.23-51.51)
Time to 1-mm ST depression	10	43.23 (33.81-52.65)	3	45.94 (40.39-51.49)	1	NA	9	42.58 (33.10-52.05)

MACE and adverse events: The sensitivity analysis confirmed the risk reductions for any MACE (0.89; 95% CI, 0.84-0.95) and MI (0.91; 95% CI, 0.84-0.98) (Table 5).

**Table 5:** Sensitivity analysis excluding studies with high risk of bias: clinical endpoints and adverse events CI, confidence interval; INOCA, ischemia with non-obstructive coronary arteries; MACE, major adverse cardiovascular events; MI, myocardial infarction; NA, not available; PCI, percutaneous coronary intervention; CABG, coronary artery bypass grafting

	Overall		>6 months			INOCA			Obstructive disease		
Outcome	Number of studies	Pooled estimate with CI	Number of studies	Pooled estimate with CI		Number of studies	Pooled estimate with CI		Number of studies	Pooled estimate with CI	
MACE											
Any MACE	4	0.89 (0.84-0.95)	4		0.89 (0.84-0.95)	NA		NA	4		0.89 (0.84-0.95)
PCI	1	NA	NA		NA	NA		NA	Nil		NA
CABG	1	NA	NA		NA	NA		NA	Nil		NA
Any Revascularization	4	0.82 (0.67-1.02)	4		0.82 (0.67-1.02)	NA		NA	4		0.82 (0.67-1.02)
MI	7	0.91 (0.84-0.98)	5		0.91 (0.84-0.99)	1		NA	6		0.95 (0.85-1.07)
Hospitalization	4	0.88 (0.75-1.03)	4		0.88 (0.75-1.03)	NA		NA	4		0.88 (0.75-1.03)
Cardiac death	4	1.02 (0.86-1.20)	4		1.02 (0.86-1.20)	NA		NA	4		1.02 (0.86-1.20)
All mortality	4	1.00 (0.86-1.16)	3		1.00 (0.86-1.16)	NA		NA	4		1.00 (0.86-1.16)
Adverse events	10	1.45 (1.25-1.67)	2		1.59 (1.17-2.17)	NA		NA	10		1.45 (1.25-1.67)

The increased risk of adverse events also remained almost similar and significant (1.45; 95% CI, 1.25-1.67). The risk reductions for MACE and MI were robust, with point estimates identical to those in the primary analysis despite the exclusion of one study assessed as high risk of bias. However, the protective effects on any revascularization PCI or CABG (0.82; 95% CI, 0.67-1.02) and hospitalization (0.88; 95% CI, 0.75-1.03) were no longer statistically significant, indicating that the high-risk studies drove these benefits in the primary analysis. The increased risk of adverse events was also confirmed to be a consistent finding.

Grading of Evidence

The pooled outcomes across clinical domains are summarized in Table 3. The GRADE certainty of evidence summary is given in Table 6.

**Table 6:** Summary of findings for ranolazine versus SoC for chronic stable angina CI, confidence interval; MACE, major adverse cardiovascular events; PCI, percutaneous coronary intervention; CABG, coronary artery bypass grafting; RCTs, randomized controlled trials; RR, risk ratio; SoC, standard of care

Outcome and follow-up	Patients (studies), N	Relative effect (95% CI)	Absolute effects (95% CI)			Certainty
			SoC	Ranolazine	Difference	
Overall MACE	13795 (five RCTs)	RR = 0.89 (0.84-0.94)	259 per 1000	231 per 1000 (218-243)	28 fewer per 1000 (from 41 fewer to 16 fewer)	⨁⨁⨁⨁ High
PCI	41164 (three non-randomized studies)	RR = 0.80 (0.55-1.18)	178 per 1000	142 per 1000 (98-210)	36 fewer per 1000 (from 80 fewer to 32 more)	⨁◯◯◯ Very low
CABG	41164 (three non-randomized studies)	RR = 0.55 (0.35-0.86)	46 per 1000	25 per 1000 (16-39)	21 fewer per 1000 (from 30 fewer to 6 fewer)	⨁◯◯◯ Very low
Any revascularization	126120 (seven non-randomized studies)	RR = 0.77 (0.64-0.91)	175 per 1000	134 per 1000 (112-159)	40 fewer per 1000 (from 63 fewer to 16 fewer)	⨁◯◯◯ Very low
Hospitalization	17255 (five RCTs)	RR = 0.87 (0.77-0.98)	273 per 1000	238 per 1000 (211-268)	36 fewer per 1000 (from 63 fewer to 5 fewer)	⨁⨁◯◯ Low
Myocardial infarction	50800 (eight RCTs)	RR = 0.91 (0.84-0.98)	90 per 1000	82 per 1000 (75-88)	8 fewer per 1000 (from 14 fewer to 2 fewer)	⨁⨁⨁⨁ High
Cardiac death	12834 (five RCTs)	RR = 1.02 (0.81-1.29)	43 per 1000	43 per 1000 (34-55)	1 more per 1000 (from 8 fewer to 12 more)	⨁⨁⨁⨁ High
All-cause mortality	13664 (four RCTs)	RR = 1.00 (0.86-1.16)	48 per 1000	48 per 1000 (41-56)	0 fewer per 1000 (from 7 fewer to 8 more)	⨁⨁⨁⨁ High
Adverse events	12534 (11 RCTs)	RR = 1.45 (1.27-1.66)	113 per 1000	164 per 1000 (144-188)	51 more per 1000 (from 31 more to 75 more)	⨁⨁⨁◯ Moderate

Ranolazine was associated with a high-certainty reduction in overall MACE and MI while showing no difference in cardiac death or all-cause mortality, also supported by high-certainty evidence. For hospitalization, the evidence suggested a reduction, but the certainty was low. For PCI, CABG, and any revascularization PCI or CABG, findings indicated possible reductions; however, the certainty of evidence was very low, largely due to reliance on non-randomized data. Ranolazine was consistently associated with an increased risk of adverse events, with the certainty of this evidence rated as moderate.

The summary of findings for ranolazine versus standard of care (SoC) for improving SAQ with GRADE is given in Table 7.

**Table 7:** Summary of findings for ranolazine versus SoC for improving SAQ CI, confidence interval; SAQ, Seattle Angina Questionnaire; SoC, standard of care; RCTs, randomized controlled trials

Outcome	Patients (studies), N	Relative effect (95% CI)	Certainty
SAQ angina frequency	9537 (nine RCTs)	4.63 (1.06-8.21)	⨁⨁◯◯ Low
SAQ angina stability	2415 (seven RCTs)	12 (1.65-22.36)	⨁◯◯◯ Very low
SAQ physical limitation	2330 (seven RCTs)	2.94 (-4.05-9.92)	⨁◯◯◯ Very low
SAQ quality of life	2417 (seven RCTs)	6.16 (-0.46-12.77)	⨁◯◯◯ Very low
SAQ treatment satisfaction	2412 (seven RCTs)	0.01 (-5.22-5.25)	⨁◯◯◯ Very low

Evidence from RCTs showed that ranolazine was associated with a modest improvement in SAQ angina frequency, supported by low-certainty evidence. For SAQ stability, the results suggested a possible benefit, but the certainty was rated as very low. For SAQ physical limitation, QoL, and treatment satisfaction, findings did not demonstrate a significant benefit, and the certainty of evidence was very low across all domains.

The evidence for ranolazine improving exercise parameters in patients with CSA is rated as high certainty (Table 8).

**Table 8:** Summary of findings for ranolazine versus SoC for improving exercise parameters CI, confidence interval; RCTs, randomized controlled trials; SoC, standard of care

Outcome	Patients (studies), N	Relative effect (95% CI)	Certainty
Exercise duration	4853 (11 RCTs)	33.15 (27.39-38.92)	⨁⨁⨁⨁ High
Time to angina	4751 (nine RCTs)	42.87 (34.23-51.51)	⨁⨁⨁⨁ High
Time to 1-mm ST depression	4772 (10 RCTs)	43.23 (33.81-52.65)	⨁⨁⨁⨁ High

This indicates strong confidence that ranolazine increases exercise duration, delays time to angina onset, and prolongs time to 1-mm ST depression compared with SoC.

Discussion

This systematic review and meta-analysis of 69 studies evaluates the efficacy, safety, and clinical role of ranolazine in CSA management. Importantly, our findings extend beyond prior reviews by demonstrating that ranolazine is associated with a statistically significant reduction in MACE and MI, suggesting a broader cardioprotective effect. We also observed substantial improvement in QoL domains, particularly in patients with microvascular disease, underscoring its targeted benefit in this subgroup. Ranolazine was further associated with reductions in some revascularization outcomes, such as PCI or CABG in pooled analyses, whereas no change was observed in cardiac death or all-cause mortality.

Patient-reported QoL outcomes mirrored functional gains. Ranolazine produced statistically significant and likely clinically relevant improvements in SAQ angina frequency, angina stability, and QoL domains, with the most pronounced effects observed in the overall pooled analysis and microvascular subgroup analysis. These symptomatic benefits are important because the relief of angina and improvement in QoL are primary treatment goals in CSA management. The treatment satisfaction and physical limitation domains showed more minor, nonsignificant pooled changes, suggesting that while patients experienced fewer and more stable angina episodes, the translation to broader physical function and treatment satisfaction may be more variable and influenced by comorbidities, baseline functional status, or expectations [2,82].

The robust and statistically significant improvements in total exercise duration (MD: 33.15 seconds), time to angina onset (MD: 42.87 seconds), and time to 1-mm ST depression (MD: 43.23 seconds) highlight the impact of ranolazine’s established efficacy. These exercise benefits were sustained across subgroup analyses for longer follow-ups (>6 months) and in patients with obstructive disease, as well as sensitivity analyses excluding studies at high risk of bias. Taken together, these findings suggest a durable anti-ischemic effect that is physiologically plausible given ranolazine’s inhibition of late inward sodium current and the consequent reduction of intracellular calcium and myocardial diastolic tension.

These results demonstrate remarkable consistency with prior high-quality meta-analyses. A 2023 review by Manolis et al. reported nearly identical improvements of 30 seconds in exercise duration, 40 seconds in time to angina, and 44 seconds in time to ST depression [83]. An earlier review by Savarese et al. (2013) also found a weighted MD of 31.9 seconds for exercise duration, further reinforcing this effect size [84]. A meta-analysis by Banon et al. (2014), which included eight RCTs, also demonstrated significant increases in total exercise duration (23.9 seconds) and reductions in weekly angina attacks [5]. Our results, drawn from a much larger pool of 69 studies, reinforce these foundational efficacy findings. More comprehensively, the Cochrane review, encompassing 17 RCTs, similarly concluded that ranolazine probably reduces angina attack frequency and slightly improves exercise tolerance, supporting its role as symptomatic therapy [85]. Where our analysis adds significant value is in the detailed synthesis of patient-reported outcomes, showing clinically meaningful improvements across multiple SAQ domains, a level of detail not fully captured in earlier reviews that focused primarily on functional parameters.

On MACE-related outcomes, pooled results were mixed. The observed reduction in overall MACE (RR: ~0.89), as well as reductions in CABG and any revascularization, such as PCI or CABG, is intriguing but should be interpreted cautiously. The apparent reduction in downstream revascularization, such as PCI or CABG, may partly reflect symptomatic improvement (fewer ischemic episodes prompting invasive evaluation) rather than a disease-modifying effect on atherosclerotic progression. Our observation of the impact of ranolazine on all-cause or cardiac mortality aligns with previous syntheses, indicating that ranolazine is as safe as other antianginal agents, with its primary benefit centered on symptomatic relief rather than mortality reduction. While prior meta-analysis of RCTs found no significant effect of ranolazine on mortality or MI, our analysis identified a small potential reduction in MI and downstream revascularization, such as PCI or CABG [14]. This divergence is likely attributable to the distinct composition of our evidence base; prior meta-analyses were primarily restricted to RCTs, whereas our analysis incorporated a broader evidence base, including large-scale observational and real-world database studies. These additional data may have provided the statistical power to detect signals not apparent in smaller, selected trial populations. Nonetheless, this finding should be considered hypothesis-generating, as it may reflect symptomatic improvement leading to fewer invasive procedures rather than an actual disease-modifying effect, a caution echoed in our primary analysis.

Safety analyses showed a higher risk of adverse events (RR: ~1.45), consistent follow-up durations, disease subgroups, and sensitivity analyses. Events were non-fatal and included gastrointestinal symptoms, dizziness, constipation, and occasional dose-limiting effects or heart rate corrected QT (QTc) interval prolongation, aligning with prior meta-analytic findings. This finding is consistent with the safety signals reported in major prior meta-analyses on the topic [85]. Substantial heterogeneity across several outcomes, especially SAQ domains and angina frequency, likely reflects differences in study populations, dosing, background therapy, outcome definitions, follow-up duration, and study design. Follow-up-based subgroup analyses explained some variability: symptom and QoL benefits were clear overall and in microvascular disease but not in studies with more than six months of follow-up, possibly due to attrition, dose changes, or more refractory patient cohorts.

Patients With Microvascular Disease

As underscored by the SAQ subgroup analysis, the benefits of ranolazine appear particularly pronounced in patients with microvascular disease. This strong clinical signal is supported by a growing body of mechanistic evidence demonstrating that ranolazine can improve objective physiological measures of microvascular function. Multiple systematic reviews have converged on this point. Ling et al. (2024) conducted a meta-analysis of eight RCTs and found that ranolazine significantly increased CFR with a standardized MD of 1.16 and also improved the myocardial perfusion reserve index [86]. An earlier review by Kofler et al. (2021) similarly concluded that ranolazine may be associated with improvements in CFR in this population [87].

While some individual trials, such as the MARINA trial [27] and a placebo-controlled trial by Bairey Merz et al. [77], did not show an overall benefit on their primary endpoints of change in CFR, both trials reported important subgroup findings. In these studies, patients with more severe baseline microvascular dysfunction (e.g., CFR < 2.0 or 2.5) did appear to derive a significant benefit from ranolazine, suggesting a targeted effect in those with the most advanced pathology. Collectively, this evidence positions ranolazine as a leading therapeutic option for the challenging microvascular disease population.

Patients With Type 2 Diabetes: A Unique Dual-Benefit Agent

Although not a primary endpoint of this meta-analysis, evidence strongly supports a meaningful role for ranolazine in patients with diabetes. The TERISA trial showed clear reductions in weekly angina despite background antianginal therapy [64]. Beyond symptom relief, ranolazine provides a metabolic benefit: a meta-analysis of over 4400 patients, a mean glycosylated hemoglobin (HbA1c) reduction of about 0.5%, consistent with findings from CARISA [27]. This combined antianginal and antihyperglycemic effect makes ranolazine an attractive option for patients with type 2 diabetes and CSA who remain symptomatic or cannot tolerate therapies that affect heart rate or blood pressure [88]. Clinicians should counsel patients about common, non-serious adverse effects and consider ECG monitoring for patients at risk of QT prolongation. Ranolazine does not reduce mortality and should not replace guideline-directed therapies known to improve prognosis.

Future research should evaluate hard clinical outcomes, such as MI, hospitalization, and revascularization, such as PCI or CABG, in well-defined groups, including those with refractory post-PCI angina, microvascular angina, and diabetes. In accordance with recent comparative evaluations of antianginal regimens, the role of metabolic modulators as add-on therapy has gained significant clinical interest. While ranolazine improves exercise duration and reduces anginal frequency through partial fatty acid oxidation inhibition, similar metabolic agents such as trimetazidine offer comparable anti-ischemic benefits via distinct yet complementary mechanisms. Integrating these comparative perspectives into the management of refractory angina provides a broader framework for optimizing individualized antianginal therapy [15]. The standardized reporting of patient-centered outcomes, especially consistent SAQ use with predefined clinically important differences, is needed to strengthen evidence synthesis. Long-term safety data, including arrhythmia risk and drug interactions, remain essential.

Strengths and Limitations of the Current Review

The primary strength of this review lies in its comprehensive scope, which incorporates many studies and evaluates a broad range of clinical and patient-reported outcomes. The rigorous methodology, including adherence to PRISMA 2020 reporting standards, prospective protocol registration with PROSPERO, and the use of robust statistical methods, enhances its validity. The detailed subgroup and sensitivity analyses provide crucial insights into the heterogeneity of treatment effects across different patient populations and help to establish the robustness of the primary findings.

The main limitations are the reliance on non-randomized studies for MACE and revascularization, which introduces confounding risks, and significant heterogeneity in SAQ domains. Additionally, the MACE reduction is a post hoc, hypothesis-generating finding from underpowered trials, while the inclusion of small, single-center studies may have influenced pooled estimates. Furthermore, while subgroup analyses based on specific clinical presentations such as microvascular angina would be of considerable interest, the current literature is limited by a scarcity of primary trials explicitly isolating these cohorts and insufficient patient-level data. Consequently, future clinical trials and individual participant data meta-analyses are necessary to evaluate the therapeutic efficacy of ranolazine in these subsets of patients.

## Conclusions

In this systematic review and meta-analysis, ranolazine was associated with meaningful improvements in angina-related symptoms, exercise tolerance, and patient-reported QoL among patients with CSA, with particularly notable benefits in those with microvascular disease. The findings also suggest potential reductions in MI, MACE, and downstream revascularization; however, these outcome signals should be interpreted cautiously because they were influenced by heterogeneity and the inclusion of non-randomized evidence. Importantly, ranolazine did not demonstrate a mortality benefit, reinforcing its role primarily as an antianginal and QoL-enhancing therapy rather than a prognostic intervention.

Overall, ranolazine may be considered a useful adjunctive treatment for patients who remain symptomatic despite standard antianginal therapy, especially when heart rate or blood pressure limitations restrict the use of other agents. Clinicians should balance its symptomatic benefits against the increased risk of generally non-fatal adverse events and consider appropriate monitoring in patients at risk for QT prolongation. Future adequately powered, long-term randomized trials are warranted to clarify its effects on hard cardiovascular outcomes, define the patient subgroups most likely to benefit, and standardize patient-centered outcome reporting.
